# Association Between Prosuicide Website Searches Through Google and Suicide Death in the United States From 2010 to 2021: Lagged Time-Series Analysis

**DOI:** 10.2196/53404

**Published:** 2024-07-26

**Authors:** Nora Clancy Kelsall, Catherine Gimbrone, Mark Olfson, Madelyn S Gould, Jeffrey Shaman, Katherine Keyes

**Affiliations:** 1 Department of Epidemiology Columbia University New York, NY United States; 2 Department of Psychiatry Columbia University New York, NY United States; 3 Department of Environmental Health Sciences Columbia University New York, NY United States; 4 Columbia Climate School Columbia University New York, NY United States

**Keywords:** pro-suicide forum, suicide, google search, social media, online forum, internet search, death, United States, suicide death, forum, analysis, association, poisoning, suffocation

## Abstract

**Background:**

The rate of suicide death has been increasing, making understanding risk factors of growing importance. While exposure to explicit suicide-related media, such as description of means in news reports or sensationalized fictional portrayal, is known to increase population suicide rates, it is not known whether prosuicide website forums, which often promote or facilitate information about fatal suicide means, are related to change in suicide deaths overall or by specific means.

**Objective:**

This study aimed to estimate the association of the frequency of Google searches of known prosuicide web forums and content with death by suicide over time in the United States, by age, sex, and means of death.

**Methods:**

National monthly Google search data for names of common prosuicide websites between January 2010 and December 2021 were extracted from Google Health Trends API (application programming interface). Suicide deaths were identified using the CDC (Centers for Disease Control and Prevention) National Vital Statistics System (NVSS), and 3 primary means of death were identified (poisoning, suffocation, and firearm). Distributed lag nonlinear models (DLNMs) were then used to estimate the lagged association between the number of Google searches on suicide mortality, stratified by age, sex, and means, and adjusted for month. Sensitivity analyses, including using autoregressive integrated moving average (ARIMA) modeling approaches, were also conducted.

**Results:**

Months in the United States in which search rates for prosuicide websites increased had more documented deaths by intentional poisoning and suffocation among both adolescents and adults. For example, the risk of poisoning suicide among youth and young adults (age 10-24 years) was 1.79 (95% CI 1.06-3.03) times higher in months with 22 searches per 10 million as compared to 0 searches. The risk of poisoning suicide among adults aged 25-64 was 1.10 (95% CI 1.03-1.16) times higher 1 month after searches reached 9 per 10 million compared with 0 searches. We also observed that increased search rates were associated with fewer youth suicide deaths by firearms with a 3-month time lag for adolescents. These models were robust to sensitivity tests.

**Conclusions:**

Although more analysis is needed, the findings are suggestive of an association between increased prosuicide website access and increased suicide deaths, specifically deaths by poisoning and suffocation. These findings emphasize the need to further investigate sites containing potentially dangerous information and their associations with deaths by suicide, as they may affect vulnerable individuals.

## Introduction

Death by suicide in the United States has increased from 10.7 per 100,000 in 2001 to 14.1 per 100,000 in 2021 [1], accelerating its public health burden. Rates of death by suicide have increased across all ages, with the greatest increases among men and those aged 10-24 years [1] While known individual-level determinants of suicide death include past nonfatal suicide attempts, depressive disorders, stressful life events, and lack of social support [2], understanding potential novel risk factors is necessary for addressing the increasing rates of suicidality.

There is evidence that exposure to content that is explicit regarding suicide, most notably after inappropriate reporting of high-profile suicide deaths, such as description of specific means, increases population rates of suicide [3,4]. It is less well understood whether novel sources of information about suicide, such as prosuicide websites with discussion forums, are related to recent changes in suicide death rates. While the majority of suicide-related websites provide resources such as helplines and encouragement to seek mental health care [5,6], not all websites share this approach. Prosuicide websites are those that promote or facilitate suicide, including advocating for an individual’s “right” to end their own life, creating communities of individuals who desire to end their own lives, as well as sharing explicit information about means of killing oneself. [7,8]. While prosuicide websites are relatively few in number [5,6] and accessibility differs by browser [9,10], search engine [11], and search terms [11,12], they can easily be reached across the world [11,13,14], and many suicide forums and related explicit information are accessible to the public without age or other restriction, and thus, are available to suicidal and otherwise vulnerable individuals [15-17].

Prosuicide website access may affect suicide risk through several different mechanisms. While some individuals find comfort or social support in the communities created on the forums, for others these sites could contribute to escalating plans to end their lives [16,18-20]. Access to the sites might also promote suicidal behavior and encourage individuals with psychiatric disorders to seriously consider or attempt suicide by normalizing suicide death or making it a more realistic option. The Interpersonal Theory of Suicide suggests that the repeated exposure to painful or fearful events may provide an increased acquired capability for suicide by reducing fear surrounding suicide and violence, and thus increase risk to an individual [21]. This construct may become pertinent when individuals engage in prolonged interactions with prosuicide websites, experiencing recurrent exposure to instances of self-harm by fellow users, alongside the dissemination of explicit details regarding methods of suicide. Users often share information about lethal means of death, which may increase the probability that an attempt will be fatal, in addition to providing supportive messages about chosen means. However, attempts through means such as firearms, which are highly lethal, are often discouraged due to the potentials for long-term disability if an attempt does not result in death, and for the greater likelihood that an attempt will be aborted when firearms are involved [22].

Previous findings are mixed on the impact of prosuicide websites. One study found an increase in suicidal ideation in those who had used the internet for suicide or mental health related reasons, including searching for suicide methods [23], whereas another found no significant association with suicidal ideation [19]. However, there remains limited information on the population level effects of these websites. In addition, some literature on the understanding of the effect of these websites may be outdated [7,8] and have limited use due to the rapidly changing landscape of contemporary internet use.

Google trend data on suicide related terms have previously been shown to be associated with the suicide rate globally [24,25], yet previous studies have not included specific terms for prosuicide websites, a potentially important addition. The present study aimed to estimate the association of the frequency of Google searches of known prosuicide web forums and content with death by suicide over time in the United States, by age as well as means of suicide death.

## Methods

### Google Searches

National monthly Google search data for names of common prosuicide websites and related terms between January 2010 and December 2021 were extracted from Google Health Trends API (application programming interface) [26] (previously the Google Flu Trends API). We queried combinations of search terms including names of prosuicide websites and related terms (exact search terms available upon request). Queries randomly select 10%-15% of total Google searches for the given month and returned the probability of a search for the given terms per 10 million searches. Geographies below the national level (eg, state or designated market area) were largely suppressed for many of the study months due to insufficient search numbers; thus, we focus on national search numbers. We requested 10 data sets and averaged the results to estimate monthly search levels.

### Deaths by Suicide

We used restricted data from the CDC National Vital Statistics System (NVSS) to identify monthly US national suicide deaths. Suicide deaths were defined as those that listed X60-X84 (*ICD-10* [*International and Statistical Classification of Diseases, Tenth Revision*]), intentional self-harm, U03, intentional self-harm resulting from terrorism, and Y87.0 (*ICD-10*), sequelae of intentional self-harm, as an underlying cause of death. Deaths were further categorized using all listed causes of death by means including: firearm (X72-X74), suffocation (X70), and poisoning (X60-X69). These means categories encompassed 92.4% of the total national suicides between 2010 and 2021; other means were not sufficiently prevalent for analysis. We also identified deaths that involved opioid overdose as a subcategory of poisoning for sensitivity analyses. Suicides involving opioids were identified as any suicide as defined above, which also listed any cause of death as T40.0-T40.4 or T40.6. We aggregated counts of death by suicide for 2 age groups: 10-24 and 25-64 years by sex and by means category. We excluded the oldest age group of 65 years and older due to potentially differing patterns of internet use. Yearly national population data by age and sex were collected from the national census bureau.

### Statistical Analysis

**Figure 1:**
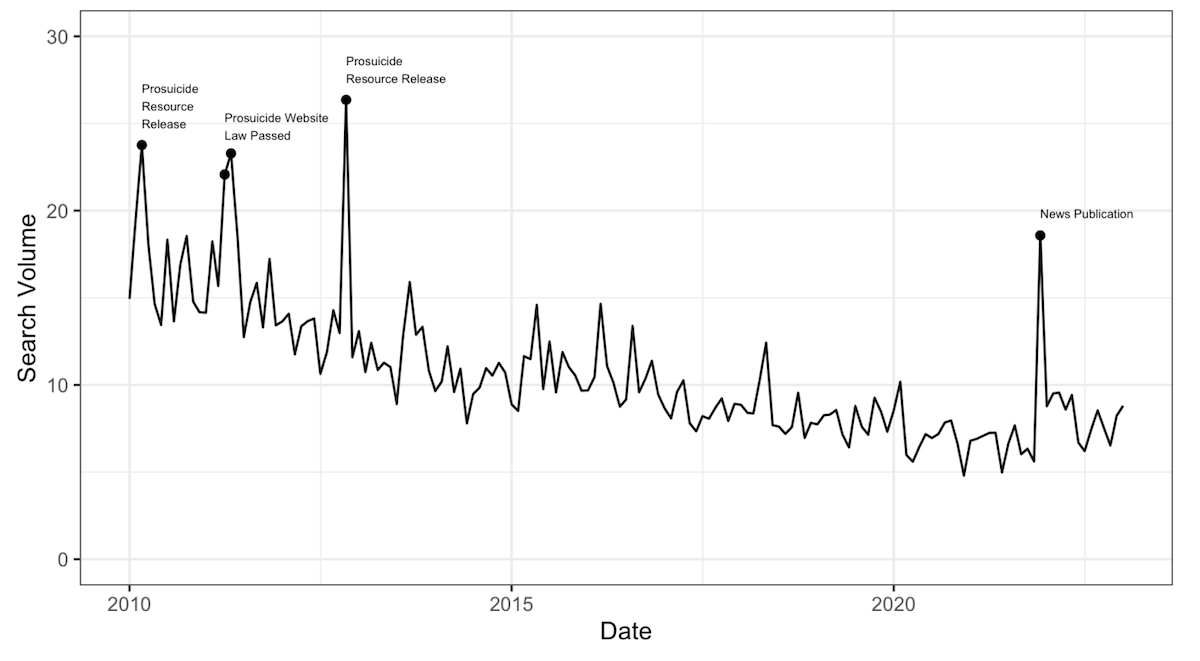
Monthly search volume of a comprehensive set of terms encompassing prosuicide websites and related terms between January 2010 and December 2021. Noted peaks were removed prior to analysis. Note prosuicide resource release refers to initial publication of a website or book with prosuicide material.

For the primary statistical analysis, we used distributed lag nonlinear models (DLNMs) [27] to estimate the lagged association between the number of Google searches on suicide mortality. DLNMs describe the association between an exposure and effect as well as a lag and effect. Monthly Google search data were used as the explanatory time series, and suicide deaths, stratified by age, sex, and means were analyzed as dependent time series. User demographics are not available for Google data. From the NVSS data, deaths were stratified by age, sex, and means, due to robust evidence that temporal and seasonal patterns of death by suicide vary by age [28,29], sex [30], as well as means [31,32]. Age was stratified into 10-24 and 25-64 years to determine differences among youth and young adults versus adult populations. These stratifications were used to evaluate potential differential associations with suicide deaths in these populations. Therefore, we investigated each dependent time series separately as we aimed to explore how searches may vary in association with different patterns of deaths. Peaks (see Figure 1) related to events such as high-profile news articles on the topic were removed from the explanatory time series.

DLNMs use a cross-basis function, which is a 2D matrix that describes the exposure-lag-response relationship using known transformations [33]. To define the cross-basis function of the Google searches, we used 3 months as the maximum lag based on previous literature examining the association between Google searches and suicide deaths [23,24,34]. We then tested linear, polynomial, and multiple degree of freedom splines for the lag-response and exposure-response. The best fitting model was selected based on the Akaike information criterion of each model [35]. In our final models, we used a linear model of the lag-response relationship and a spline with 3 degrees of freedom to model the exposure-response relationship.

We then used the cross-basis function of searches in a generalized linear Poisson model controlling for month of the year (to account for seasonality) using a spline with 4 degrees of freedom, as well as an offset term for population. We used 0 searches as a reference point. Analyses were stratified by age at death, sex, and cause of death. Results with 95% CIs that did not include 1, as computed using a normal approximation of the estimators, were considered statistically significant. Results only discuss statistically significant results.

### Sensitivity Analyses

To test whether results were robust to alternative model specifications, we also used (seasonal) autoregressive integrated moving average [(S)ARIMA] models to evaluate the correlation between the explanatory and dependent time series following the steps described in Tran et al 2017. We first fit a (S)ARIMA model to the explanatory time series. We auto-selected this model using the *forecast* package in R, which considers the possibility of a seasonal component. This model was evaluated using plots of the standardized residuals, autocorrelation functions of the residuals, and Q-Q plots of the standardized residuals. The residuals from this model were considered the prewhitened explanatory time series. The same model was then fit to the dependent time series, and the residuals from this model were considered filtered dependent time series. The correlation between the prewhitened explanatory time series and the filtered dependent time series was then evaluated using a cross-correlation function. A positive correlation at a negative lag would indicate that an increase in searches occurred prior to an increase in deaths, and vice versa for a negative correlation. Like the DLNM models, we used 3 months as our maximum lag and stratified by age, sex, and cause of death.

To estimate whether results from the DLNM models were robust to the reporting period, we conducted sensitivity analyses including shorter time periods. Two time periods were selected that were an equal number of months, from January 2010 to December 2015, and January 2016 to December 2021. The same process for the distributed lag models was followed separately for each of these time periods. In addition, given that opioid deaths have significantly increased in the United States in the past 10 years [36], we wanted to evaluate whether the results may be spuriously produced due to increasing trends in suicide deaths that involved opioids. Thus, to evaluate a potential differential association between searches and opioid deaths, lagged associations between suicide deaths from only opioid poisoning, and only nonopioid poisoning, were also evaluated.

We also repeated the DLNM analyses using Google search terms that were related to suicide but not specific to prosuicide websites. The search terms that were combined and used for this analysis included “suicide -squad,” “suicides,” and “suicidality,” which were drawn from Tran et al 2017 [24]. The minus sign in “suicide -squad” indicates removing the searches with the word squad, in order to avoid references to the movie. We considered other search terms that are reliably associated with suicide deaths, especially those about specific methods. Given that such terms may direct individuals to suicide website forums, we chose more general terms. Analysis for these terms followed the same DLNM procedure as the primary search terms as described above.

### Ethical Considerations

The New York State Institute Institutional Review Board determined that this research was exempt from human participants review (protocol AAAS5604).

## Results

Monthly Google search data were collected for the time period between January 2010 and December 2021 (Figure 1). There were 4 distinct peaks during this period, during the months of March 2010, April-May 2011, November 2012, and December 2021. These peaks corresponded to releases of prosuicide resources, passing of relevant laws, and a news article, and were removed from the time series for analysis.

**Figure 2:**
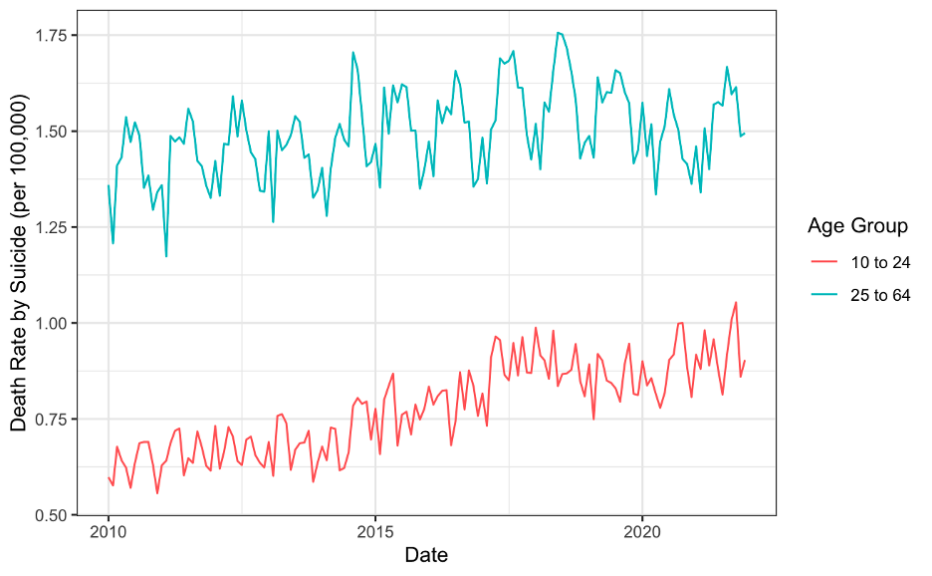
Monthly rates of death by suicide per 100,000 between January 2010 and December 2021 for age groups 10-24 and 25-64 years.

Deaths by suicide varied by age group, and rates of death by suicide were higher in 2021 than in 2010 for all age groups. Individuals aged 10-24 years had the lowest rates of death by suicide (Figure 2, Table 1).

**Table 1:** Demographics and means of deaths by suicide across age between 2010 and 2021.

		10-24 (N=71,961), n (%)		25-64 (N=362,344), n (%)
**Sex**				
	Male	57,053 (79.3)	277,968 (76.7)	
	Female	14,908 (20.7)	84,376 (23.3)	
**Means^a^**				
	Firearm	33,516 (46.6)	169,661 (46.8)	
	Suffocation	27,301 (37.9)	102,951 (28.4)	
	Poisoning	6397 (8.9)	66,476 (18.3)	

^a^Means are not mutually exclusive.

### DLNM Models

#### Youth Aged 10-24 Years

**Figure 3:**
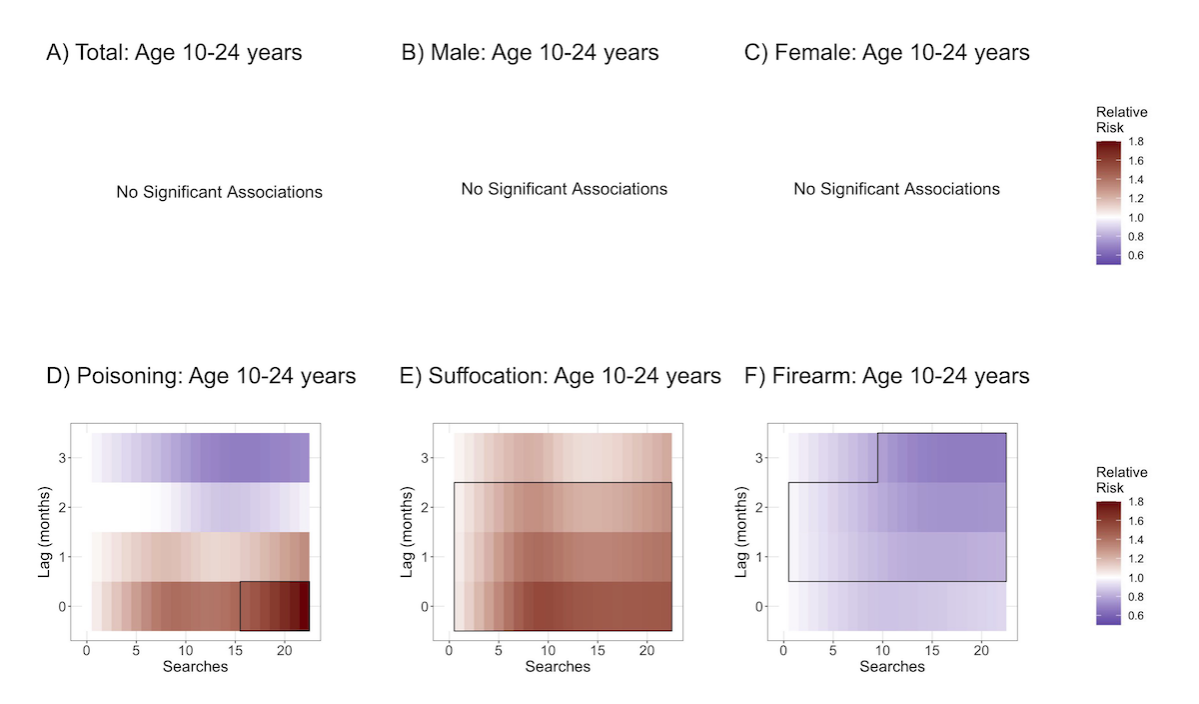
Log of relative risks of an increase from 0 searches for (A) total suicides for those aged 10-24 years, (B) male suicides among (10-24 years), (C) female suicides (10-24 years), (D) poisoning suicides (10-24 years), (E) suffocation suicides (10-24 years), and (F) firearm suicides (10-24 years). Boxed areas are statistically significant.

Figure 3 shows a plot of the association between search rates and deaths among those aged 10-24 years by sex and method at lags of 0 months (concurrent), 1 month, 2 months, and 3 months; areas in red are those with a positive association (increases in searches were associated with higher suicide rates), and areas in blue are those with a negative association (increases in searches were associated with lower suicide rates). A positive association at a lag of 1 month means that an increase in searches was associated with an increase in deaths by suicide in the following month. Associations with the total deaths among youth and young adults aged 10-24 years as well as deaths by sex showed no significant associations (Figures 3A-3C).

Associations with youth intentional poisoning deaths showed a positive association at lag 0 with a maximum relative risk of 1.79 (95% CI 1.06-3.03) at the maximum search volume of 22 searches per 10 million as compared to no searches, indicating that the risk of suicide was 1.79 times higher in months with 22 searches per 10 million as compared to 0 searches. There were no significant association at lags 1, 2, or 3 (Figure 3D).

For deaths involving suffocation, there was a significant positive association at lags 0, 1, and 2 months with maximum relative risks as compared to no searches of 1.55 (95% CI 1.23-1.96) at a search volume of 9 per 10 million, 1.43 (95% CI 1.26-1.61) at a search volume of 9 per 10 million, and 1.31 (95% CI 1.09-1.59) at a search volume of 8 per 10 million, respectively, with the strongest association at lag 0 (Figure 3E).

Associations with firearm deaths showed a significant negative association at lags 1, 2, and 3 months with minimum relative risks as compared to no searches for lags 1, 2, and 3 months of 0.80 (95% CI 0.71-0.91) at a search volume of 14 per 10 million, 0.74 (95% CI 0.64-0.85) at a search volume of 15 per 10 million, and 0.67 (95% CI 0.50-0.90) at a search volume of 17 per 10 million, respectively. The reverse association was strongest at a lag of 3 months (Figure 3F).

#### Adults Aged 25-64 years

**Figure 4:**
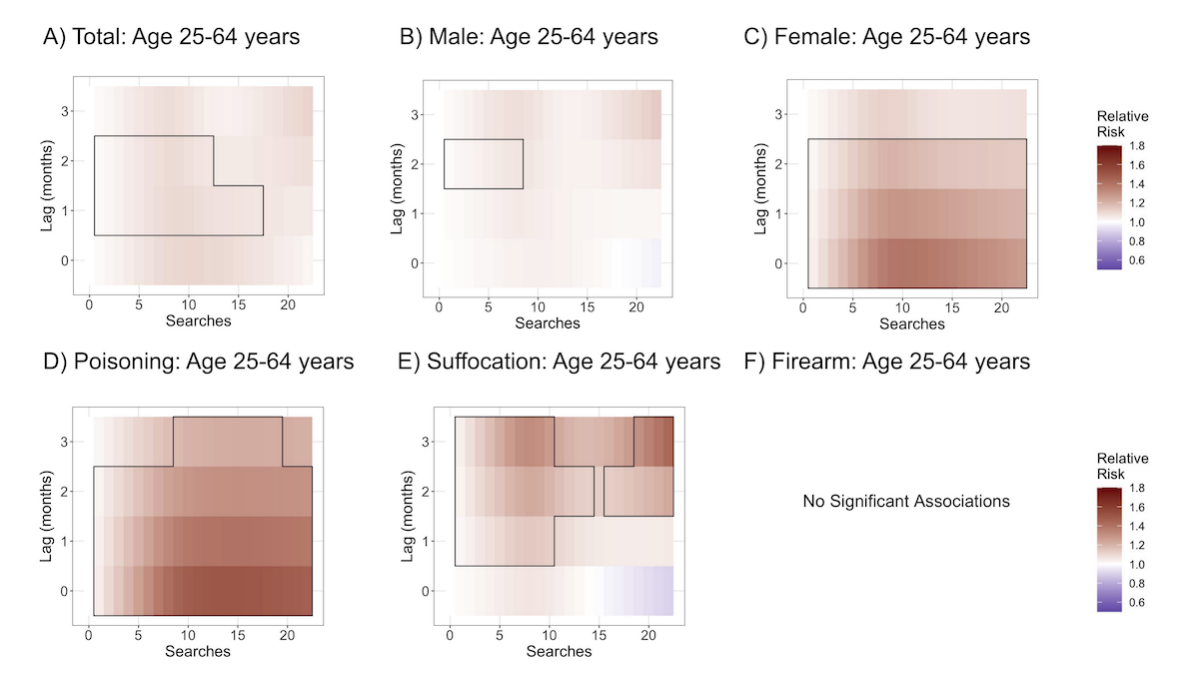
Log of relative risks of an increase from 0 searches for (A) total suicides for those aged 25-64 years, (B) male suicides (25-64 years), (C) female suicides (25-64 years), (D) poisoning suicides (25-64 years), (E) suffocation suicides (25-64 years), and (F) firearm suicides (25-64 years). Boxed areas are statistically significant (*P*<.05).

The association between search rates and deaths by suicide for adults aged 25-64 years old shown in Figure 4 differed from associations found for youth. Search association with total adult deaths was statistically significant at lags 1 and 2 months, with a maximum relative risk as compared to no searches of 1.10 (95% CI 1.03-1.16) at a search volume of 9 per 10 million and 1.09 (95% CI 1.02-1.16) at a search volume of 8 per 10 million, respectively, indicating that the strongest signal for adults aged 25-64 years was for increased search rates in a given month associated with increased suicide deaths the following month and 2 months later.

There were few significant associations with male deaths, with significant associations only at lag 2, with a maximum relative risk as compared to no searches of 1.06 (95% CI 1.00-1.11) at a search volume of 8 per 10 million; however, there was a significant association with female deaths at lags 0, 1, and 2 months with maximum relative risks as compared to no searches of 1.40 (95% CI 1.19-1.63) at a search volume of 10 per 10 million, 1.29 (95% CI 1.19-1.40) at a search volume of 10 per 10 million, and 1.20 (95% CI 1.10-1.31) at a search volume of 9 per 10 million, respectively. The strongest association was at lag 0.

Associations with intentional poisoning deaths were positive at all lags with maximum relative risks as compared to no searches of 1.50 (95% CI 1.29-1.75) at a search volume of 14 per 10 million, 1.40 (95% CI 1.30-1.51) at a search volume of 15 per 10 million, 1.31 (95% CI 1.21-1.43) at a search volume of 15 per 10 million, and 1.23 (95% CI 1.03-1.45) at a search volume of 16 per 10 million for lags 0, 1, 2, and 3, respectively. The strongest association was at lag 0.

Associations with deaths by suffocation were similarly positive at all lags; however, associations at lag 0 did not reach significance. Maximum relative risks as compared to no searches were 1.14 (95% CI 1.03-1.28), 1.23 (95% CI 1.09-1.38), and 1.44 (95% CI 1.05-1.97) for lags 1, 2, and 3 months, respectively, all at a search volume of 8 per 10 million.

Associations with firearm deaths were not significant at any lag.

### Sensitivity Analyses

SARIMA model results were distinct between youth and adults. For youth, similar to the DLNM analysis, SARIMA analysis showed a consistent peak at 0 lag for total youth deaths by suicide, including among men and women, and for suffocation. However, unlike the DLNM models, there was a peak at 0 lag in firearm deaths. Deaths by poisoning were the only group without a statistically significant positive association with search rates at lag 0; however, estimates show a positive trend. All groups of youth deaths except females also had a significant negative association at either lag 2 or lag 3 (Figure S1 in Multimedia Appendix 1). Unlike DLNM models, when investigating associations for adults aged 25-64 years, there was no evidence of an association for any group at any lag (Figure S2 in Multimedia Appendix 1).

The patterns of association in the 2 subperiods were mostly consistent with the results for the entire record (Figure S3 in Multimedia Appendix 1). The time period between 2016 and 2021 had stronger associations between searches and means-specific deaths for both youth and adults. However, the association with total deaths was stronger in the earlier time period for youths.

Opioid-related suicide deaths accounted for (21,441/85,399, 25.1%) of intentional poisoning deaths between 2010 and 2021, and included the use of synthetic opioids, heroin, methadone, codeine, and morphine. The pattern of the relationship with search rates for prosuicide websites was similar between poisoning suicides due to opioids and those that were not opioid-related; however, for youth, the magnitude of the association between search rates and deaths was only significant for opioid-related deaths as compared to those that were not opioid-related (Figure S4 in Multimedia Appendix 1).

Repeating the DLNM analyses using Google search terms that were not specific to prosuicide websites identified no significant associations between search rates and deaths by suicide for female youth and adults, deaths by poisoning for youth and adults, or deaths by suffocation for adults. In addition, the significant associations with deaths were largely negative, except for the association between searches and deaths by suffocation for youth aged 10-24 years (Figure S5 in Multimedia Appendix 1).

## Discussion

During months when searches of prosuicide websites increased, there were more deaths by intentional poisoning and suffocation, among both youth and adults. However, for youth, we also observed that increased searches were associated with fewer suicide deaths by firearm at lags of 1, 2, and 3 months. These findings are suggestive of an association between searches and suicide deaths, and warrant further exploration. There are multiple potential explanations for this association, both causal and noncausal. The findings could be the evidence of a causal relationship with these sites influencing individuals to take their own life. On the other hand, increases in searches for such sites could be a marker of an increase in population suicidality, although results of sensitivity analyses for other suicide-related terms suggest otherwise.

We observed distinctly different patterns among adolescents and young adults in associations with means of death. Among these age groups, while there were positive concurrent associations between searches and deaths by suffocation and poisoning, there were negative time-lagged associations with suicide deaths by firearm. Messaging common on prosuicide websites may explain this pattern. While there has been little academic study of the content of suicide websites, our review of the posts consistently noted that deaths by firearm were often discouraged and firearm self-injury was discussed as having a high risk of long-lasting physical disability (as has been noted by others [37]). In contrast, poisoning and suffocation were discussed more often and were “encouraged.” Associations may also be higher with deaths by poisoning and suffocation due to more complex procedures required for death by these means; thus, increased access to specific information may have a greater impact on lethality.

Within specific means, the associations differed by age group. There was a positive association at all lags for all age groups with deaths by suffocation; however, for youth, associations were of greater magnitude. In contrast, associations between searches and death by poisoning were of greater magnitude for adults than youth. These patterns may be due to differing availability of means for youth, as suffocation is the most frequent nonfirearm means used by youth presumably due to greater ease of access to these means [38-40]. Poisoning, which tends to involve more complex procedures and difficult to obtain materials, is less common among youth [41].

Sensitivity analyses showed that there was some variation in the association of prosuicide site search rates with suicide by time period. Most associations were closer to the null between 2010 and 2015 as compared to between 2016 and 2021. This may be due to a change in the material shared on these sites over time, or different sites being created or shut down. Data were too sparse to conduct COVID-19–specific sensitivity analyses between 2020 and 2021; however, this is a question that requires further investigation.

Sensitivity analyses also showed that for youth, the associations were stronger for opioid-related intentional poisoning deaths compared to nonopioid-related intentional poisoning deaths; however, there was no corresponding difference for adults. While speculative, it should be noted that deaths from opioids in the United States have increased rapidly during this study period, including deaths designated as suicides among youth [36,42]. Individuals dependent on opioids have higher suicide rates than the general population, and thus suicidal individuals with access to opioids, especially highly lethal synthetic opioids, might have been more vulnerable to prosuicide messages. In addition, as adults are more likely to use multiple medications than youth [43], the effect of searches on adult opioid overdoses may be lower due to the availability of other medications.

Results also show that the patterns of association differed in models based on general suicide search terms from prosuicide website specific search terms. While more research is needed to fully investigate these findings, significant associations between searches of general suicide-related terms and deaths by suicide were largely negative, indicating a potential protective effect of seeking general suicide information using search engines, which could be due to the majority of web-based sources providing recovery and support resources [6], and showing that the current findings are indicative of a distinct effect of prosuicide sites. This also suggests that the association between searches and deaths by suicide is not solely a marker of an increase in population level suicidality due to differing patterns in associations.

For youth, the ARIMA models showed similar results for most groups as the DLNM models. They showed a positive association between searches and deaths by suicide at 0 lag, with a negative association at a 2- or 3-month lag. They varied most from the DLNM models when estimating association with deaths by firearms; DLNM models showed only negative associations, while ARIMA models showed a pattern similar to other deaths by suicide. In addition, ARIMA models showed no association for adults, whereas DLNM models showed significant associations for most groups. These differences in results may be due to DLNM allowing for fitting of a nonlinear model to the associations, as well as differing methods for controlling for long-term variability.

Given the dangerous information they provide, efforts to limit accessibility to prosuicide websites and information have already taken place in several countries. The original domain name of one of the most well-known prosuicide websites was blocked or restricted in Australia, Italy, and Germany [44,45]. In the United States, following a high-profile news article of this website and associated deaths, there were legal actions proposed including criminal actions toward the owner of the website. Tech companies such as Google and Bing were also urged to limit accessibility [45]. Companies including Google and Meta have already taken some precautionary measures surrounding availability of such information, such as automatically providing information on suicide prevention resources based on search terms and allowing users to flag posts about self-harm or suicide [46,47]. However, these measures have limited efficacy [48]. Meanwhile, this website and other similar forums continue to be openly accessed with no effective age barriers and give explicit instructions on how to die by suicide [11,13,14]. It is well understood that while mentioning suicide in general and inquiring about suicidal ideation does not increase risk of suicide, discussion of specific suicide means and sensationalizing suicide deaths can increase the risk [3,4].

Our results suggest that there is a population level association between search rates of prosuicide websites and deaths by suicide. However, there are several limitations to this study. Google search volume does not indicate the population conducting these searches, limiting the interpretation of age or sex-specific associations. In addition, by using Google search volume to estimate access to these websites, we likely underestimated the true website seeking behavior, as there are other ways of accessing these sites that are not captured in Google searches, including via other search engines and direct links from other web pages. Further, Google search volume data were suppressed at the state level; thus, we were not able to account for state-level differences in potential covariates such as access to lethal means. While we were able to attend to some potential confounders such as increased lethality of the opioid supply, and included a seasonal term in the model to account for seasonal patterns, other potential confounders may be considered in future studies, including factors varying over time such as economic factors and media exposure. This study also used fatal suicides as a measure of suicidality; however, prosuicide websites may be associated with chosen means and subsequent fatality of an attempt rather than rates of attempts, which should be investigated in further research.

Our results emphasize potentially pernicious population-level effects of prosuicide websites on suicide risk among youth and adults. Although preliminary, the findings suggest that searches of these websites are associated with deaths by suicide among youth and adults and underscore the need to regulate or close these sites.

## Appendix

Multimedia Appendix 1 Additional figures.

## Abbreviations

API: application programming interface
ARIMA: autoregressive integrated moving average
DLNM: distributed lag nonlinear model
ICD-10: International and Statistical Classification of Diseases, Tenth Revision
NVSS: National Vital Statistics System
(S)ARIMA: (seasoned) autoregressive integrated moving average

## Data Availability

Data used for these analyses are not publicly available in order to limit dissemination of names of prosuicide websites, but are available from the corresponding author on reasonable request.
