# Supplementary material for: Association Between Prosuicide Website Searches Through Google and Suicide Death in the United States From 2010 to 2021: Lagged Time-Series Analysis
Source: J Med Internet Res. 2024 Jul 26;26:e53404. doi: 10.2196/53404 (PMC11316151; doi:10.2196/53404)

Multimedia Appendix 1

S1. Results of ARIMA model analyses showing correlations between searches and a) Total Suicides for those ages 10-24, b) Male Suicides 10-24, c) Female Suicides 10-24, d) Poisoning suicides 10-24, e) Suffocation suicides 10-24, f) Firearm suicides 10-24. Dotted blue lines indicate statistical significance (*P* < 0.05).


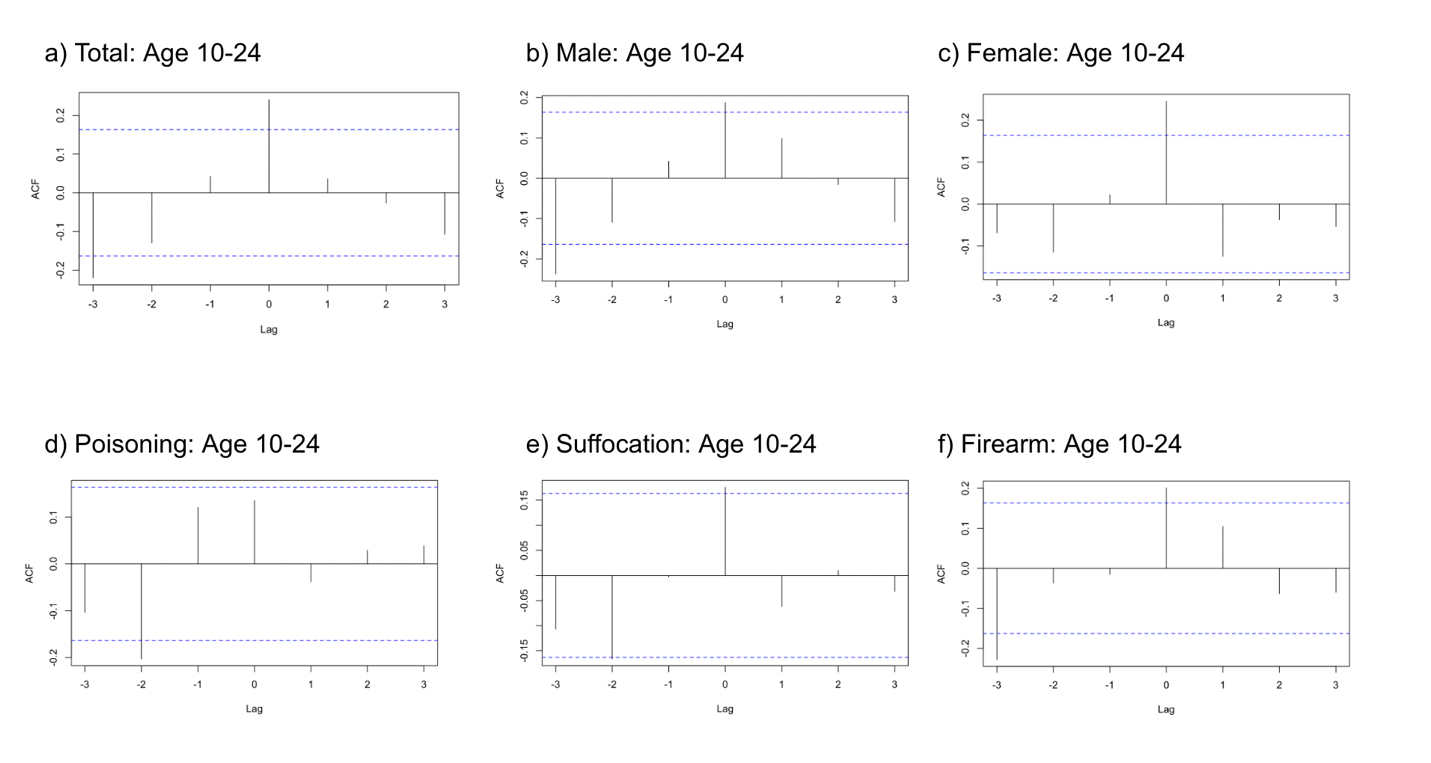


S2. Results of ARIMA model analyses showing no statistically significant correlations between searches and a) Total Suicides for those ages 25-64, b) Male Suicides 25-64, c) Female Suicides 25-64, d) Poisoning suicides 25-64, e) Suffocation suicides 25-64, f) Firearm suicides 25-64. Dotted blue lines indicate statistical significance (*P* < 0.05).


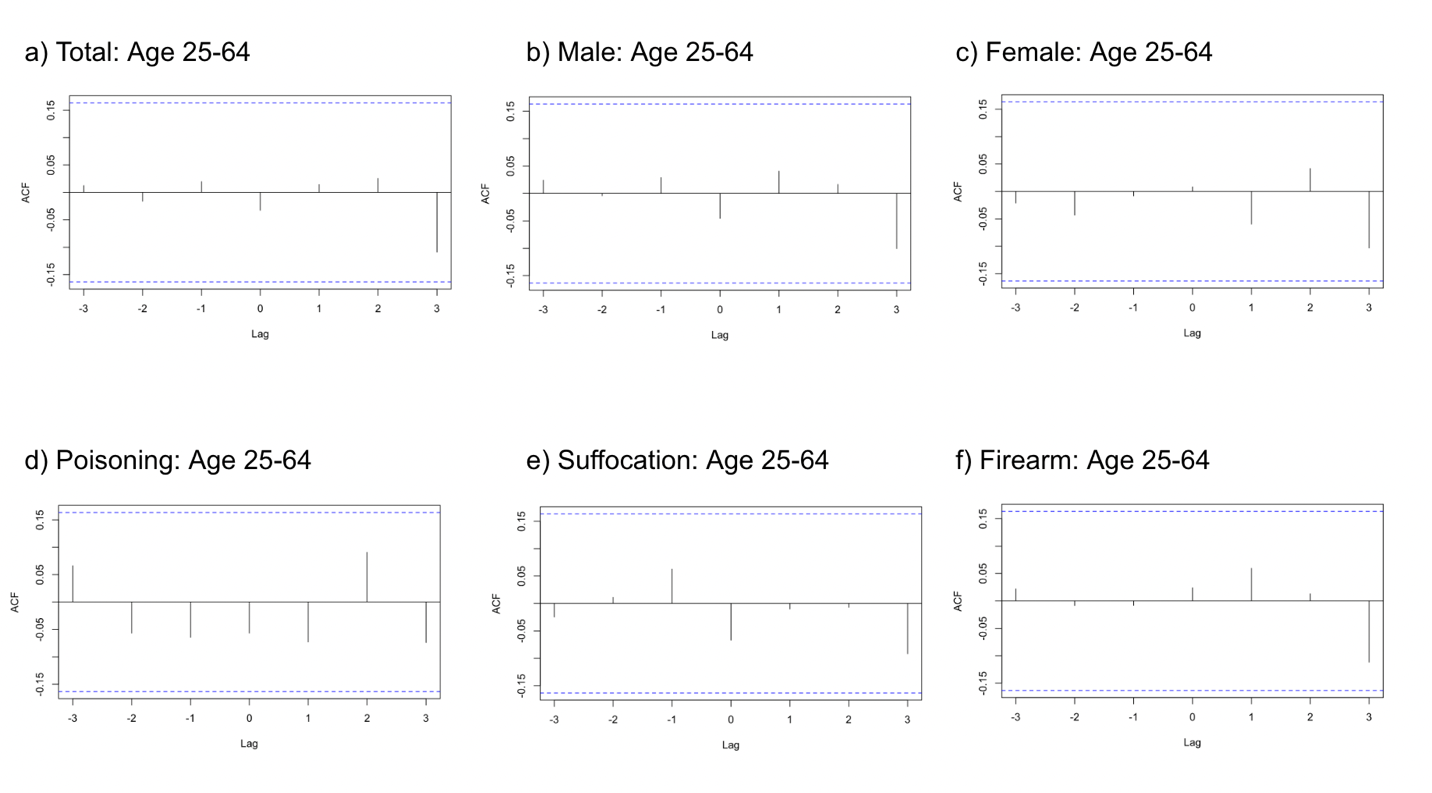


S3. Comparison of associations during the entire time period (2010-2021) with two different time periods (2010-2015, 2016-2021) for deaths total deaths by age group, as well as deaths by age group and means. Boxed areas are statistically significant.


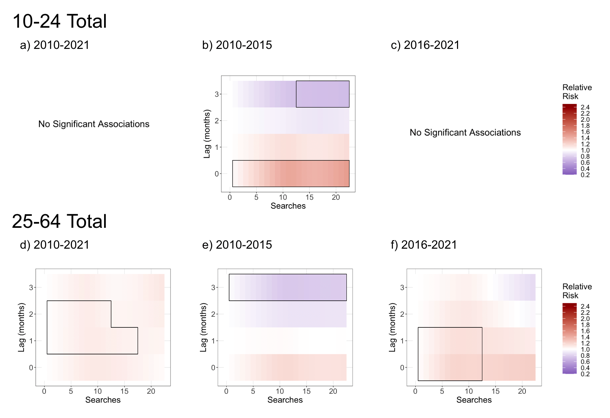


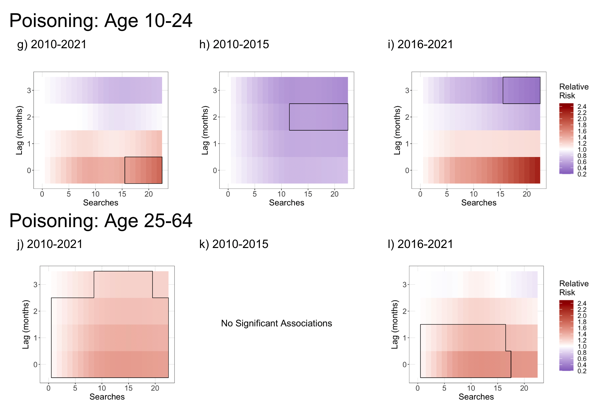


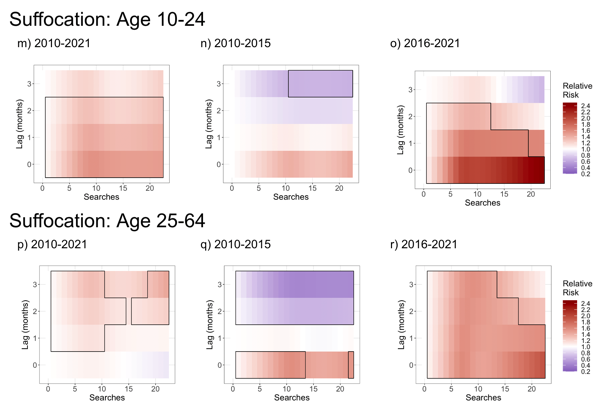


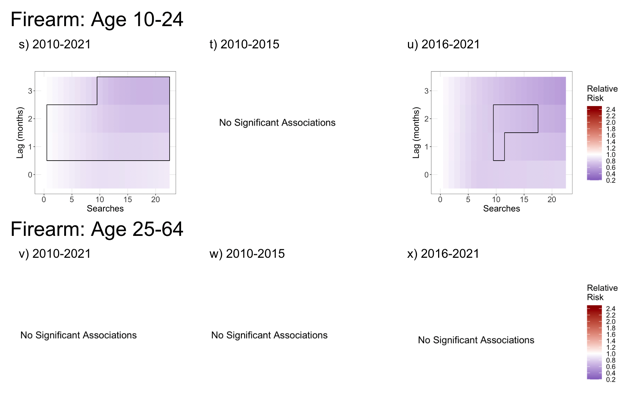


S4. Sensitivity analysis to investigate differences between associations between searches and all intentional poisoning deaths, and intentional poisoning deaths that were not opioid related. Note the difference in scales between the age groups. Boxed areas are statistically significant.


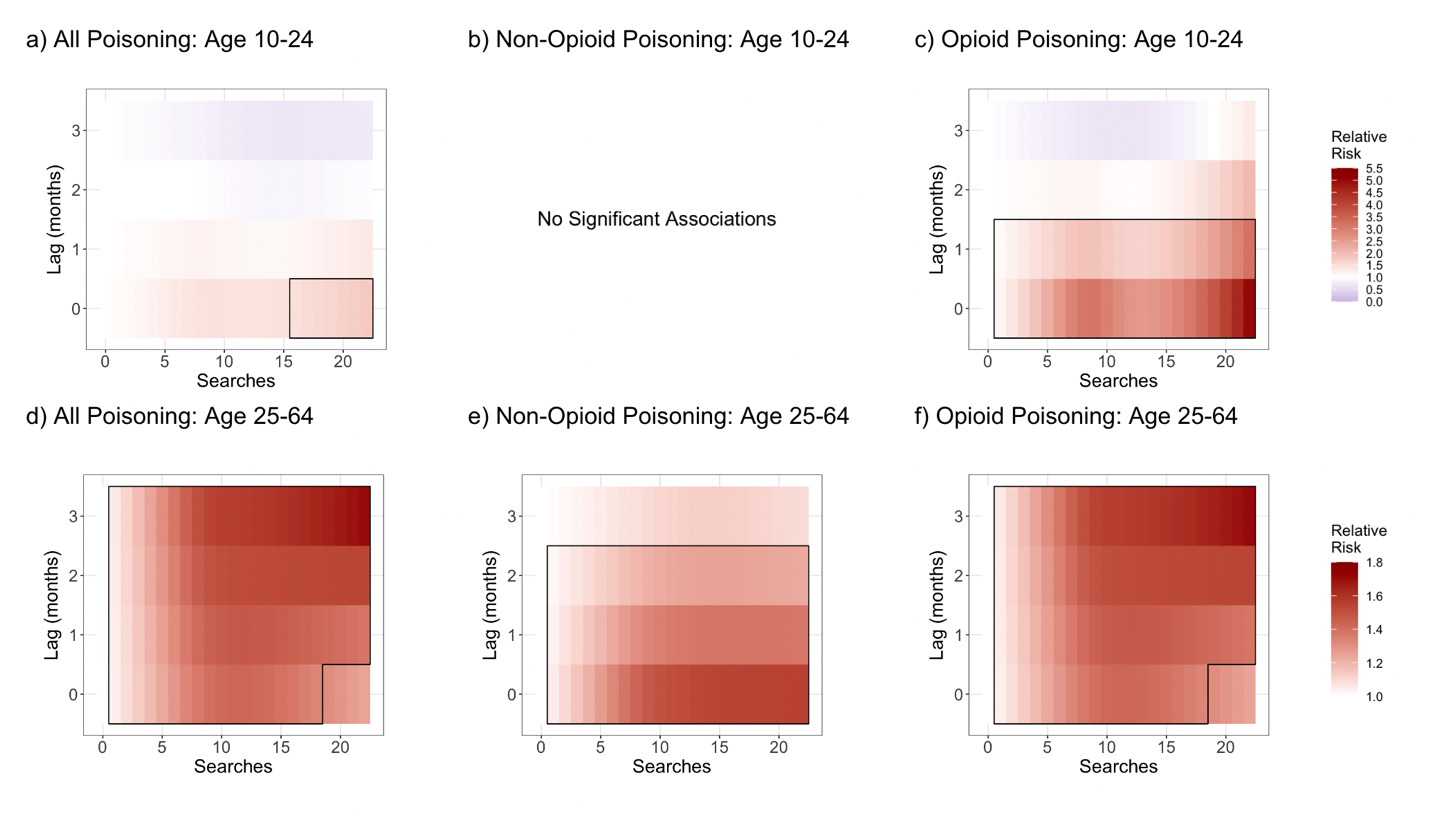


S5. Sensitivity analysis using DLNM methods to investigate associations between general suicide search terms and suicide deaths, between 2010 and 2021. Boxed areas are statistically significant (*P* < 0.05).


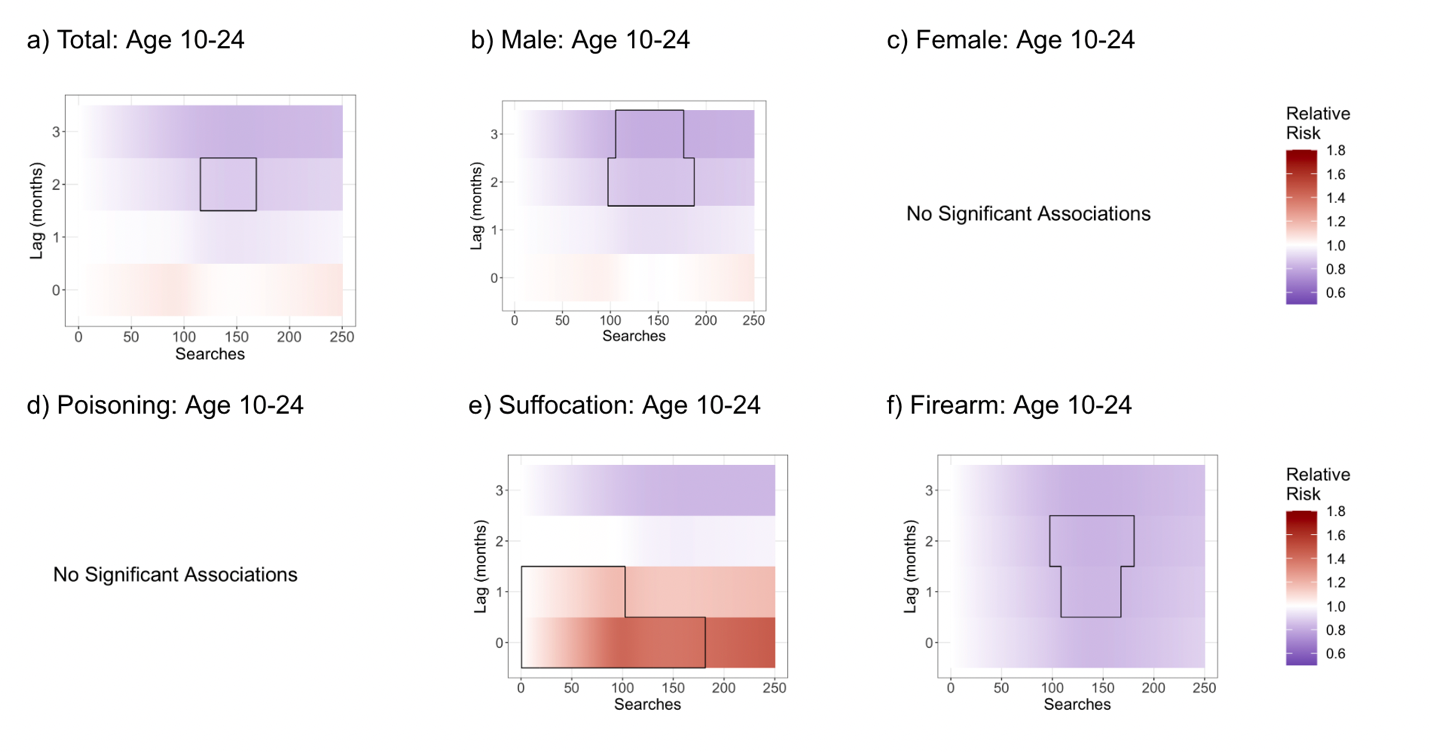


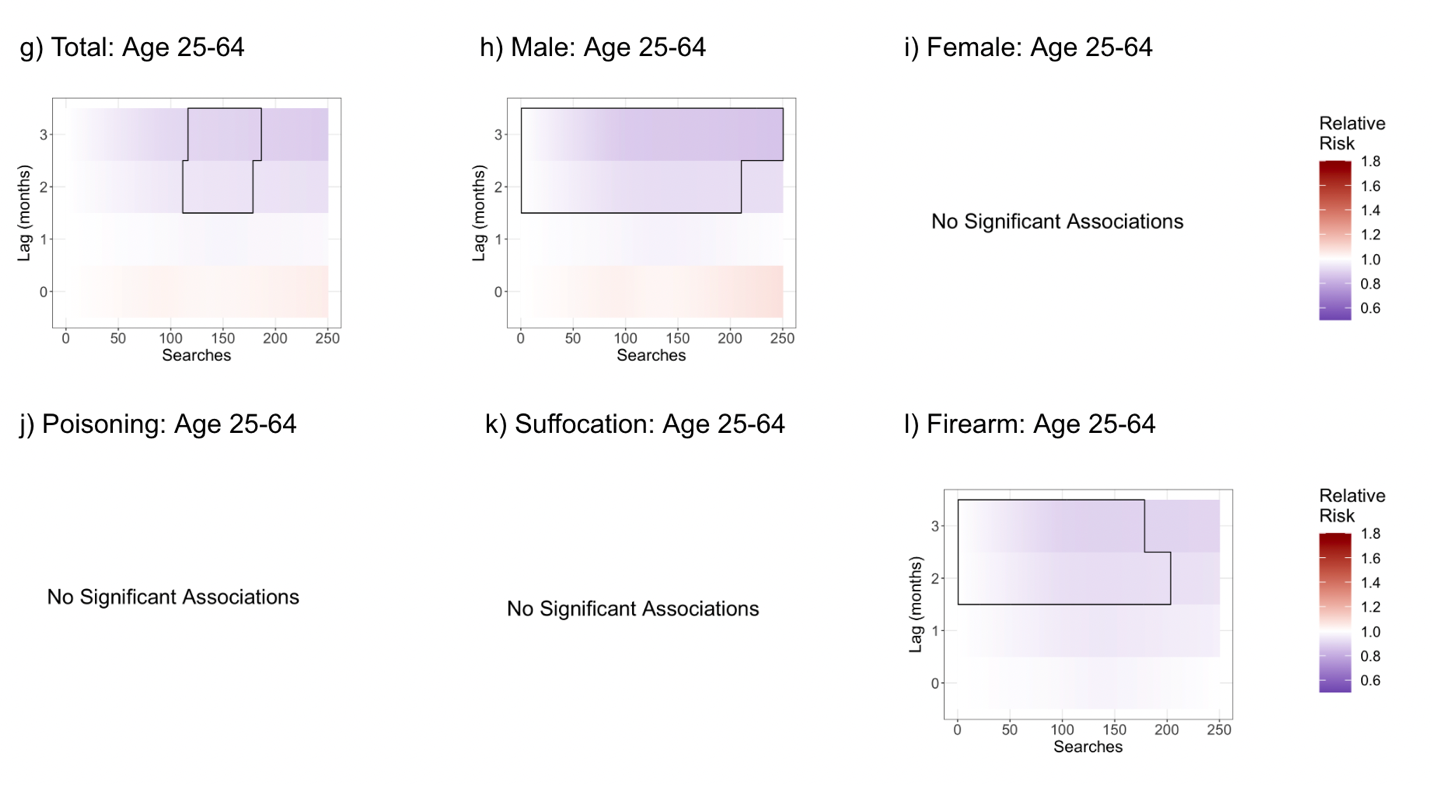

Supplement: Multimedia Appendix 1 [file jmir_v26i1e53404_app1.docx]
